# Aging Strong: Promoting Resilience Through Optimism, Purpose, and Social Connections

**DOI:** 10.1093/geroni/igab046.687

**Published:** 2021-12-17

**Authors:** Ellen Wicker, James Schaeffer

## Abstract

Resilience is defined as the ability to adapt and cope with circumstances in a way that empowers one to emerge stronger, thrive, and incorporate lessons learned. Resilience as a trait can be learned and modified and have a significant impact on healthy aging. UnitedHealthcare (UHC) and AARP Services, Inc. (ASI) are committed to the health and well-being of participants in UHC’s Medicare Supplement insurance plans, recognizing that health and wellness need to be promoted on a holistic level to ensure successful aging. In this effort, an initiative titled Aging Strong 2020 was developed to promote health, well-being, and increase resilience by focusing on the key individual pillars of enhanced purpose in life, social connectedness, and optimism. To accomplish this goal, a series of eight interventions over three years were created and delivered, with a focus on the key pillars in order to improve clinical and psychological health outcomes and participants’ satisfaction with health care. This symposium will specifically discuss efforts related to the Aging Strong 2020 program. First, we will describe the prevalence and outcomes of the pillars in a large national survey. Next, key challenges and successes in recruitment and retention for the various interventions will be highlighted, followed by overall findings from the eight interventions targeting the pillars. Finally, qualitative findings on participant experience as a result of participation will be discussed. Results from these initiatives demonstrate that interventions designed to improve well-being among older adults contribute to the holistic model of health.

